# Identification, Evaluation, and Treatment of Patients with Hereditary Cancer Risk within the United States

**DOI:** 10.1155/2013/260847

**Published:** 2013-12-22

**Authors:** Deborah Cragun, Tuya Pal

**Affiliations:** Department of Cancer Epidemiology, Moffitt Cancer Center, 12902 Magnolia Drive, Tampa, FL 33612, USA

## Abstract

Recognizing the importance of identifying patients at high risk for inherited cancer predisposition, the United States Preventive Services Task Force (USPSTF) has outlined specific family history patterns associated with an increased risk for BRCA mutations. However, national data indicate a need to facilitate the ability of primary care providers to appropriately identify high risk patients. Once a patient is identified as high risk, it is necessary for the patient to undergo a detailed genetics evaluation to generate a differential diagnosis, determine a cost-effective genetic testing strategy, and interpret results of testing. With identification of inherited predisposition, risk management strategies in line with national guidelines can be implemented to improve patient outcomes through cancer risk reduction and early detection. As use of genetic testing increasingly impacts patient outcomes, the role of primary care providers in the identification and care of individuals at high risk for hereditary cancer becomes even more important. Nevertheless it should be acknowledged that primary care providers face many competing demands and challenges to identify high risk patients. Therefore initiatives which promote multidisciplinary and coordinated care, potentially through academic-community partnerships, may provide an opportunity to enhance care of these patients.

## 1. Introduction

**Figure 1:**
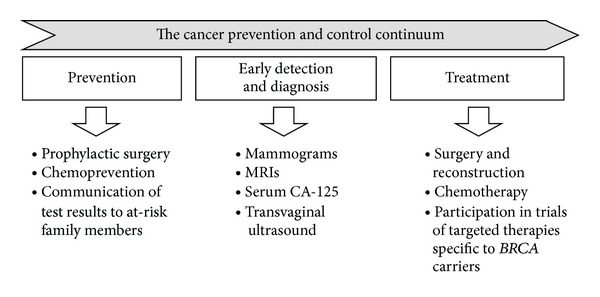
Management of *BRCA* mutation carriers across the cancer prevention and control continuum.

As the field of clinical cancer genetics has matured, more community-based and primary care providers are identifying and testing individuals who are at high risk for hereditary cancer syndromes or referring high-risk patients to a genetics professional [1, 2]. Genetic testing for hereditary cancer has implications across the cancer prevention and control spectrum, from risk assessment to diagnosis, to treatment (as illustrated for *BRCA* mutation carriers in Figure 1). Using the *BRCA1* and *BRCA2* (*BRCA*) genes discovered almost 2 decades ago as examples [3, 4], there are now evidence-based management guidelines that improve patient outcomes through cancer risk reduction and early detection [5–7]. Specifically, for those women with a *BRCA* mutation, interventions such as prophylactic mastectomy and oophorectomy reduce incidence of breast cancer and ovarian cancer by over 95% and 80%, respectively [8–14]. In recognition of the increasing importance of identification and management of inherited cancer risk, the 2009 ACOG practice bulletin indicated that routine obstetrics and gynecology (OB/GYN) practice [15] should include: (1) recognition of high risk patients based on personal and family history (“high risk” for hereditary breast and ovarian cancer has been defined by national organizations and summarized in Tables 1 and 2) [16, 17]; (2) integration of risk assessment, testing, and results interpretation; and (3) implementation of a management plan based on risk stratification. In contrast to OB/GYN, other primary care subspecialties have not developed practice guidelines that support taking a comprehensive role in genetic testing and management of patients at high risk for hereditary cancers. Nevertheless, all primary care providers are critical in the identification and referral of high risk patients. Furthermore, primary care providers can play an important role by encouraging and/or facilitating appropriate risk management options and screening recommendations for patients who are diagnosed with hereditary cancer syndromes.

## 2. Identification of Patients at High Risk

Identifying patients at high risk for hereditary breast and ovarian cancer as well as other hereditary cancer syndromes falls within a broader standard of care that involves: (1) obtaining a comprehensive and complete family history and updating it on a routine basis; (2) giving patients appropriate information based on the family history collected in order for them to make informed decisions about their care; and (3) recording the patient encounter within the medical record [18]. Nevertheless, meeting this standard of care can be challenging for several reasons. First, practitioners are faced with many competing demands that force them to do more in less time. Second, the lack of clear definitions regarding the meaning of “high risk” for hereditary cancer and what constitutes a comprehensive and complete family history poses additional challenges. Lastly, determining which patients are at high risk has also proven challenging.

Although there are no standardized criteria, the United States Preventive Services Task Force (USPSTF) has outlined specific family history patterns associated with an increased risk for *BRCA* mutations (“high risk women”), as listed in Table 1. According to the USPSTF, high risk women should be referred for genetic counseling and evaluation for *BRCA* testing (which is a Grade B recommendation) [19]. Furthermore, USPSTF recommends against routine referral for genetic counseling or testing for women “without an increased-risk family history” (grade D recommendation) because potential harms outweigh benefits. However, these guidelines do not consider women with a personal history of breast or ovarian cancer; the guidelines fail to outline how high risk should be determined, and they fail to address risks for other hereditary cancer syndromes.

Regarding provider ability to stratify risk level, a nationally representative vignette-based survey of primary care providers (defined to include family practitioners, general internists, and obstetricians/gynecologists) suggests that many average-risk women are offered genetic counseling and/or *BRCA* testing [20]. Furthermore, although obstetricians/gynecologists (OB/GYN) were more likely than the other two physician subspecialties to almost always offer genetic counseling or testing in a high risk scenario, over 32% of OB/GYNs did not do so. Another large survey of primary care physicians indicated that all OB/GYN subspecialists and most internists and family practitioners were aware of *BRCA* testing [21]. Furthermore, over 60% of OB/GYN and approximately 22% of internists and family practitioners had ordered at least one *BRCA* test in the last year. However, even among physicians who had ordered testing, less than a third consistently recognized high-risk and low-risk family history patterns [21]. More recently, results of a study of over 2500 women based in a large health system indicated that 90% of high risk women who met USPSTF guidelines for consideration of *BRCA* testing shared this information with their primary care physicians; however, less than 20% had been referred for genetic counseling and only 8% had undergone testing [22]. These findings highlight the lack of provider ability to correctly stratify women at high versus average risk for *BRCA* mutations and emphasize the need to improve health care infrastructure and clinician education to realize the population level benefits of *BRCA* testing.

Interestingly, another study of primary care providers (also defined to include family medicine, internal medicine, and OB/GYN) affiliated with a single insurance carrier indicated that 83% reported routinely assessing for hereditary cancer risk. However, only 33% reported that they take a full, three-generation pedigree [23], which is needed to perform a risk assessment per standard of care guidelines [24, 25]. Thus, it is not surprising that studies to assess provider collection of family history based on chart reviews have suggested a lack of information to adequately assess risk in a substantial number of charts [26, 27]. Specifically, review of ambulatory medical records of 734 family practice patients indicated documentation of family history of cancer in 97.8% of records; however there was insufficient information to adequately assess hereditary cancer risk in over 2/3 of charts [26]. Findings from another review of over 10,000 oncology charts of patients with either breast or colorectal cancer indicated insufficient documentation of family history details needed for hereditary cancer risk assessment in the majority of patients [27]. Moreover, referral for genetic counseling and/or testing was documented in only 52.2% and 26.4% of high risk patients with breast and colorectal cancer, respectively. These data further serve to illustrate the need for intervention efforts to promote collection of sufficient family history information and enhanced ability for risk stratification.

Identification of those at high risk for *BRCA* mutations is becoming increasingly important as the Department of Health and Human Services, the Department of Labor, and the Department of Treasury have recently indicated that genetic testing for breast cancer is to be covered as a preventive service under the US Affordable Care Act (ACA) for high risk patients according to USPSTF guidelines. This classification will likely broaden access to *BRCA* testing, but only if high risk patients are appropriately identified so they may be offered a detailed genetics evaluation to discuss testing options. Health history tools [28], referral screening tools [29, 30], and/or interactive preventive health records [31] may help in overcoming barriers to the collection and use of family history information in primary care, thereby aiding in the identification of high risk patients who should be offered further evaluation.

## 3. Evaluation and Testing of High Risk Patients

Following the identification of high risk patients, it is recommended that genetic risk assessment is conducted as part of a genetic counseling session, during which patient consent is obtained in cases where testing is appropriate. Genetic counseling and testing for hereditary breast and ovarian cancer have evolved to include multiple health care professionals (e.g., master's trained genetic counselors, nurses, and physicians) and specialties (e.g., gynecology, genetics, and internal/family medicine) based in a variety of settings (e.g., community and academic) [32–34]. Several professional organizations have outlined guidelines regarding content of the risk assessment session and informed consent [24, 25, 35]. These guidelines generally include collection of a 3-4 generation pedigree, generation of a differential diagnosis and genetic testing strategy, discussion of the risks for hereditary cancer syndromes, benefits and limitations of testing, insurability-related concerns, and an overview of management options for hereditary cancer. In the case of possible hereditary breast and ovarian cancer, several risk prediction models are available for estimating the probability that a *BRCA* mutation will be identified [36, 37]. When testing is performed, the ordering practitioner is responsible for interpreting and explaining the results to the patient, providing information regarding appropriate cancer screening recommendations and risk management options, discussing implications for other family members, and encouraging patients to share this information with at-risk family members.

Providers require a high level of proficiency in cancer genetic risk assessment in order to deliver high quality services, which has been the subject of a number of recent articles. Specific issues identified include inappropriate or incomplete testing and misinterpretation of test results by both patients and clinicians, leading to inappropriate cancer screening/prevention recommendations and psychological issues [38–42]. On the surface, the sharing of genetic test results may appear basic. However, when the intricacies are examined, the knowledge needed for dissemination of results, which includes positive, negative (i.e., “true negative” and “uninformative”), and indeterminate (i.e., variant of uncertain significance (VUS)), can become quite complex [43]. In addition to case series [38, 39], results of a few provider-based surveys of primarily nongenetics professionals who order *BRCA* testing have suggested opportunities to improve cost-effectiveness of testing practices and quality of services [41, 42]. Specifically, a statewide survey of Texas physicians indicated that many order unnecessary additional *BRCA* testing in the context of a VUS result [42]. Our recent survey of Florida-based healthcare providers revealed similar findings [41]. Additional findings from our survey included lack of practitioner recognition of the need to perform *BRCA* large rearrangement testing when comprehensive BRCAnalysis did not detect a mutation and concerns that practitioners may interpret an uninformative negative *BRCA* test result to mean that the patient is not at high risk for developing cancer, despite a strong family history. Furthermore, a strong family history may indicate that testing for other hereditary cancer syndromes is warranted; therefore practitioners must also recognize additional patterns of hereditary cancer beyond those associated with *BRCA* mutations.

The need to recognize other hereditary cancer syndromes may become less important given the expected increase in cancer panel-based testing, whereby multiple genes for various hereditary cancer syndromes are tested simultaneously. Due to reduced costs associated with new sequencing technologies, there are already cases when it may be more cost-effective to order panel-based testing as opposed to sequentially testing for single genetic conditions. However, interpreting results from panel-based testing adds complexity due to factors such as: questionable or uncertain clinical relevance of testing for moderate penetrance genes and the higher rate of inconclusive results, due to variants of uncertain significance (VUS) [44, 45].

These issues and complexities serve to highlight the importance of providing multidisciplinary care and genetics expertise to high risk patients. Although research is needed to compare the effectiveness of various genetic service delivery models that exist within the U.S. [46, 47], one model for providing multidisciplinary care is through development of community-academic partnerships to promote the involvement of genetic professionals in the care of community-based patients [48, 49]. Furthermore, the development of these types of care models has great potential to facilitate continuing education and give community patients the opportunity to participate in clinical research in this rapidly evolving field [49, 50]. For example, we have a statewide effort to support research, education, and outreach initiatives focused on *BRCA* genetic counseling and testing through the Inherited Cancer Registry (ICARE) for which external peer-reviewed funding was secured in 2010 [49]. The ICARE initiative leverages a state mandate to reach the citizens of Florida and provide access to high quality cancer care. The ICARE team works with partners (called “ICARE partners”) across the state and beyond to offer clinical expertise and research opportunities at an NCI-designated comprehensive cancer center. Through networking with our ICARE partners, providing access to a genetic counselor for general questions and other directed learning opportunities and educational resources/materials over the last 3 years, we have established contact with 127 healthcare providers from 89 external sites who offer *BRCA* testing. An objective measure of our success is illustrated by the level of interest in accessing our provider-targeted resources, which include our web-based bimonthly genetics case conferences with community partners to provide education and discussion of challenging cases. In fact, attendance at our case conferences has grown yearly and over the last year has averaged 12 or more unique sites participating at each conference. In addition to our educational and outreach efforts, ICARE partners refer high risk patients to our research registry to provide the research link, which has in turn contributed to the tremendous growth of our registry since initiation of the grant in summer 2010. The distribution of biyearly newsletters (available at http://inheritedcancer.net/), focused on clinical and research advances pertaining to those with inherited cancer, has been received with much enthusiasm by registry participants and ICARE partners. Patients have even reported sharing this information with their other health care providers who are not involved with ICARE. These types of efforts provide opportunities to facilitate education and outreach with the objective of widely disseminating information about the rapid advances in the specialized field of inherited cancer predisposition. These efforts are expected to lead to information that can improve the delivery of cancer genetic services in the state of Florida and may serve as a model for other states.

## 4. Management of High Risk Patients

Individuals with *BRCA* mutations are estimated to have a 60–70% lifetime risk of breast cancer [51–55] and up to a 40% risk of developing ovarian cancer [51, 53]. Once an individual is identified as a *BRCA* mutation carrier, the objective is for the individual and their family members to benefit from knowing this information through prevention, early detection, and treatment as depicted in Figure 1. Outcomes of *BRCA* carriers following risk reducing surgeries indicate benefits of both prophylactic mastectomy and salpingooophorectomy in lowering cancer risks as well as cancer-specific and all-cause mortality [12]. Specifically, current data suggests breast cancer risk reduction of over 90% with prophylactic mastectomy [8–12, 56, 57] and reduction in ovarian cancer of approximately 80% with prophylactic oophorectomy [13, 14]. As for breast surveillance, although MRI is the most sensitive screening option, current evidence supports the benefits of both mammography and breast MRI annually in *BRCA* mutation carriers [7, 58–61]. Finally, ovarian cancer surveillance remains a challenge and has not been shown to be effective in detecting early stage ovarian cancers [62]. Tamoxifen is the main chemopreventive agent evaluated in the medical context, and its role in primary prevention of breast cancer remains unclear [63], although efficacy of 50–70% reduction in the risk of contralateral breast cancer has been reported [64–66].

In order to promote best practices in a rapidly advancing field, the National Comprehensive Cancer Network (NCCN) publishes annual practice guidelines for *BRCA* mutation carriers [16]. These guidelines outline recommended risk management and surveillance options based on the most current literature and expert opinion [67, 68]. Current cancer surveillance guidelines issued by NCCN for *BRCA* mutation carriers include strong recommendations for annual mammogram and annual breast magnetic resonance imaging (MRI) for breast cancer and consideration of biannual serum CA-125 level and transvaginal ultrasound for ovarian cancer. Surgical options include bilateral prophylactic mastectomy and salpingooophorectomy. Tamoxifen as a breast cancer risk reducing agent is included in the guidelines for practitioners to consider, acknowledging there is limited data currently available to determine its efficacy.

Despite the availability of NCCN practice guidelines, results of recent provider-based surveys suggest there are opportunities to improve adherence to these guidelines [40, 41]. Specifically, a statewide survey of Texas physicians indicated that management recommendations of *BRCA1* mutation carriers were not consistent with NCCN guidelines among many providers [40]. More recently, results of our Florida-based survey of healthcare providers indicated use of additional screening tests (i.e., breast ultrasound) which are not part of the NCCN guidelines and can lead to unnecessary heath care costs [41].

Overall, the current data suggests that there remains an opportunity to enhance the delivery of care for those with *BRCA* mutations through targeted efforts to improve clinician education and adherence to national practice guidelines. Additionally, these issues will become of increasing importance as expectations for coordinated care are implemented through the affordable care act, with the objective of improving cost-effectiveness and quality of care.

## 5. Risk Management and Patient Safety Considerations

It has become increasingly apparent that identifying patients at high risk for hereditary cancer predisposition is becoming standard of care for primary practitioners when considering both the USPSTF and ACOG recommendations that outline the following expectations: (1) to obtain a comprehensive family history which is periodically updated; (2) to give patients the required information based on family history needed to make educated decisions about their healthcare; and (3) to document discussion with the patient within the medical record [18]. With regard to documentation, items that are important to include are (1) what was discussed, (2) the reasoning behind the discussion, and (3) the outcome of the discussions. For example, for a patient with a strong family history of breast and ovarian cancer, it would be important to document that, based on this strong family history, the patient's risk of inherited breast cancer may be clarified through genetic counseling and testing, which in turn may inform targeted medical management strategies. Furthermore, it is important to document whether the provider (1) recommends a referral to a genetics professional for genetic counseling or (2) if genetic counseling and testing are performed by the patient's provider himself. In the latter case when the provider orders the genetic test, it is important to document necessary discussion elements preceding testing including that informed consent has been secured [24, 25, 35]. Informed consent entails outlining all treatment options along with risks and benefits of each of the options. In the event that the patient is not interested in pursuing genetic counseling and/or testing following discussion with this provider, it is also important to document informed refusal. This refers to the documentation that the provider has made the appropriate recommendation and the patient has chosen not to proceed with the recommendation. In the current landscape, new allegations are increasingly occurring in the area of failure of the “duty to inform” or “duty to warn” [18, 69, 70]. These allegations refer to the failure to identify a patient at risk for inherited cancer predisposition, which may have led to increased surveillance or risk-reducing surgeries that have great potential to improve patient outcomes through early detection or primary prevention of cancer. This type of situation highlights the need for documentation of a patient's refusal of testing as well as explanations of cancer risks and available risk management options.

Ultimately, advances in genomic medicine will lead to additional patients who have disorders with a recognizable genetic component and will require specific medical management. Thus, healthcare professionals who perform genetic testing without adequate proficiency in genetics-based care put their patients at risk of not receiving the best available care. Furthermore, recent literature has suggested that physicians appear to be the most vulnerable group in terms of liability risks related to genetic technologies, and with growth of the field of genetics, the number of lawsuits is expected to increase substantially [69, 70].

## 6. Conclusions

Knowledge gains over the next decade will lead to advances in understanding disease pathogenesis due to genetic variation present within the human genome. Primary care practitioners are uniquely positioned to (1) identify patients at high risk for inherited cancer predisposition; (2) promote the appropriate use of genetic counseling and testing services; and (3) help facilitate risk management strategies in line with best practice guidelines. As we continue to make great progress in the arena of genetics-based care, an enormous amount of patient-specific genetic and genomic information will place a substantial burden on individual practitioners, who will require ongoing education in a broad range of areas [71]. Ultimately, genomic technologies provide unprecedented opportunities to understand health and disease; however translation of these advances to benefit patient care will require greatly improved provider proficiency in genetics and a collaborative multidisciplinary approach.

## Figures and Tables

**Table 1:** USPSTF increased-risk family history patterns.

Non-Ashkenazi Jewish women	
Two first-degree relatives with breast cancer (at least 1 under age 50 at diagnosis)	
Three or more first- or second-degree relatives with breast cancer at any age	
Both breast and ovarian cancer among first- or second-degree relatives	
A first-degree relative with bilateral breast cancer	
Two or more first- or second-degree relatives with ovarian cancer at any age	
A first- or second-degree relative with both breast and ovarian cancer	
Male relative with breast cancer	
Women of Jewish ancestry	
Any first-degree relative (or 2 second-degree relatives on the same side of the family) with breast or ovarian cancer	

**Table 2:** NCCN guidelines (version 2.2013): criteria for further genetic risk evaluation for breast/ovarian cancer.

An affected individual with one or more of the following	An unaffected individual with a family history of one or more of the following
A known mutation in a breast cancer susceptibility gene within the family	A known mutation in a breast cancer susceptibility gene within the family
Early age of onset (≤50 years)	≥2 breast primaries in a single individual
Triple negative (ER-, PR-, HER2-) breast cancer	≥2 individuals with breast primaries on the same side of the family
Two breast cancer primaries in a single individual	
Breast cancer at any age and 1 of the following:	≥1 ovarian cancer primary from the same side of the family
≥1 close blood relative with breast cancer <50 y	
≥1 close blood relative with epithelial ovarian cancer (any age)	First- or second-degree relative with breast cancer ≤45 y
≥2 close blood relatives with breast cancer and/or pancreatic cancer at any age	
From a population at increased risk	
≥1 family member on same side of family with a combination of breast cancer and ≥1 of following: pancreatic cancer, aggressive prostate cancer, sarcoma, adrenocortical carcinoma, brain tumors, endometrial cancer, leukemia/lymphoma, thyroid cancer, dermatologic manifestations and/or macrocephaly, hamartomatous polyps of GI tract, and diffuse gastric cancer	≥1 family member on same side of family with a combination of breast cancer and ≥1 of following: pancreatic cancer, aggressive prostate cancer, sarcoma, adrenocortical carcinoma, brain tumors, endometrial cancer, leukemia/lymphoma, thyroid cancer, dermatologic manifestations and/or macrocephaly, hamartomatous polyps of GI tract, and diffuse gastric cancer
Ovarian cancer	Male breast cancer
Male breast cancer	
